# Esophageal Pressure Measurement in Acute Hypercapnic Respiratory Failure Due to Severe COPD Exacerbation Requiring NIV—A Pilot Safety Study

**DOI:** 10.3390/jcm11226810

**Published:** 2022-11-17

**Authors:** Alexandru Tudor Steriade, Mihai Gologanu, Roxana Silvia Bumbacea, Stefan Nicolae Bogdan, Dragos Bumbacea

**Affiliations:** 1 Department of Pneumology & Acute Respiratory Care, “Elias” Emergency University Hospital, 011461 Bucharest, Romania; 2 Department of Cardio-thoracic Pathology, “Carol Davila” University of Medicine and Pharmacy, 020021 Bucharest, Romania; 3 National Institute for Research and Development in Microtechnologies, 077190 Bucharest, Romania; 4 Department of Allergy, “Carol Davila” University of Medicine and Pharmacy, 020021 Bucharest, Romania; 5 Department of Cardiology, “Elias” Emergency University Hospital, 011461 Bucharest, Romania

**Keywords:** acute respiratory failure, esophageal pressure, noninvasive ventilation (NIV)

## Abstract

Esophageal pressure (Pes) measurements could optimise ventilator parameters in acute respiratory failure (ARF) patients requiring noninvasive ventilation (NIV). Consequently, the objectives of our study were to evaluate the safety and accuracy of applying a Pes measuring protocol in ARF patients with AECOPD under NIV in our respiratory intermediate care unit (RICU). An observational cohort study was undertaken. The negative inspiratory swing of Pes (ΔPes) was measured: in an upright/supine position in the presence/absence of NIV at D1 (day of admission), D3 (3rd day of NIV), and DoD (day of discharge). A digital filter for artefact removal was developed. We included 15 patients. The maximum values for ∆Pes were recorded at admission (mean ∆Pes 23.2 cm H_2_O) in the supine position. ∆Pes decreased from D1 to D3 (*p* < 0.05), the change being BMI-dependent (*p* < 0.01). The addition of NIV decreased ∆Pes at D1 and D3 (*p* < 0.01). The reduction of ∆Pes was more significant in the supine position at D1 (8.8 cm H_2_O, *p* < 0.01). Under NIV, ∆Pes values remained higher in the supine versus upright position. Therefore, the measurement of Pes in AECOPD patients requiring NIV can be safely done in an RICU. Under NIV, ∆Pes reduction is most significant within the first 24 h of admission.

## 1. Introduction

Esophageal pressure (Pes) can be used as a surrogate for pleural pressure. Measuring Pes has been used to study pathophysiological mechanisms in acute respiratory failure and ventilator dependency in invasive mechanical ventilation. Consequently, monitoring Pes has been shown to improve ventilator parameters (i.e., improving PEEP adjustment and patient–ventilator interaction, optimizing protective lung ventilation parameters and weaning) in acute respiratory failure (ARF) patients on invasive mechanical ventilation [1,2]. Yet, it is rarely used in clinical practice because practitioners view this method as too complex and time-consuming. In addition, technical difficulties could be related to the insertion and proper positioning of the esophageal catheter, patient tolerance, obtaining accurate readings, and interpretation of measurements [3].

Most published studies in this field were done on patients undergoing invasive mechanical ventilation [1]. Only limited data are available regarding its applications in patients with ARF undergoing noninvasive ventilation (NIV). Most of these studies are mainly focused on the physiological observations regarding the effect of MV on respiratory mechanics [4,5,6,7]. Although the potential of this method to improve outcomes is large, the use of Pes measurement as a tool to guide NIV management has only recently been proposed, and thus, data are still scarce [8,9,10].

Our study aimed to evaluate the safety and accuracy of applying a Pes measuring protocol in conscious patients with ARF due to AECOPD under NIV in a respiratory intermediate care unit (RICU).

## 2. Materials and Methods

Our team conducted an observational cohort study in an academic center’s respiratory intermediate care unit between 2017 and 2018. We included consecutive patients with AHRF due to severe COPD exacerbation that were non-invasively ventilated with BPAP with a hybrid volume assured pressure support (VAPS) mode.

### 2.1. Noninvasive Ventilation

NIV was initiated and managed in compliance with international guidelines [2]. More specifically, we applied NIV in patients with COPD exacerbation who, despite one hour of controlled oxygen therapy, inhaled bronchodilators and systemic steroids at admission and fulfilled one of the following criteria: pH lower than 7.35 with pCO_2_ higher than 47 mmHg; dyspnea at rest with a respiratory rate higher than 23 breaths/min; use of accessory respiratory muscles; or paradoxical abdominal breathing. Exclusion criteria were: refusal of NIV or deep hypercapnic coma; facial deformity; upper gastrointestinal bleeding; tracheal stenosis; acute ischemic heart disease; and psychomotor agitation requiring sedation or urgent intubation due to cardiac or respiratory arrest [11].

Patients were ventilated for as long as possible to maintain a pH > 7.35 within the first 24 to 48 h. In the case of clinical and arterial blood gas improvement, we gradually decreased NIV duration within the following days. NIV weaning was successful if the patient did not require NIV for 48 h. We defined NIV failure as the need for endotracheal intubation or in-hospital death.

Patients were ventilated using Trilogy 100 ventilators (Phillips Respironics, Murrysville, PA, USA) with oro-nasal masks. The initial ventilator settings were BPAP S/T VAPS mode with IPAP min/max 16/30 cm H_2_O, EPAP 6–8 cm H_2_O, respiratory rate back-up 16/min, and desired tidal volume 5–6 mL/kg. These initial settings were adjusted when necessary by experienced senior staff.

### 2.2. Pes Measurement Procedure

We considered patients eligible for Pes measurement if blood gas and clinical signs improved 30 to 60 min after NIV initiation.

The Pes measuring device consisted of an esophageal balloon catheter (Cooper Surgical Inc., Trumbull, CT, USA) and a Braebon Ultima Dual Airflow Pressure Sensor Model 0580 (Braebon Medical Corporation, Ottawa, ON, Canada) connected to a Picolog 1000 data acquisition system (Pico Technology, Cambridgeshire, UK). The Pes signal was recorded using the Picolog Recorder software (Pico Technology, Cambridgeshire, UK).

After performing local anesthesia with lidocaine 2%, the esophageal balloon catheter was inserted into the nose and progressively advanced to the hypopharynx. We asked the patient to perform swallowing maneuvers with the head kept in slight anteflexion. The appropriate insertion length was estimated using the Stanford formula for esophageal manometry: 0.228 × height (cm) [12]. A suitable catheter position was confirmed by performing an end-expiratory occlusion maneuver while simultaneously measuring airway and esophageal pressure changes during an inspiratory effort (Baydur’s occlusion test) [1].

The Braebon pressure sensor allowed us to record Pes and airway pressure (Paw). Paw was recorded at the mask level. For each recording, the balloon catheter was inflated with 1 mL of air. Once in position, the catheter was maintained in the same place until discharge or was removed earlier at the patient’s request. Oral alimentation was not restricted.

Pes was measured daily in 30 min recording sessions. We made the first recording on admission. Depending on the patient’s position and ventilatory support, each recording included four stages: in the upright position with and without NIV support and in the supine position with and without NIV support. After NIV weaning, the recordings included only two stages: upright and supine. The esophageal tube did not influence the ventilation schedule or medication received during hospitalization.

### 2.3. Analyzed Parameters and Objectives

Pes was recorded during each measurement session while the patient was in an upright or supine position, with the presence or absence of NIV.

The variation of esophageal pressure, i.e., the negative inspiratory swing of Pes (ΔPes) and the mean ΔPes for each recording stage, were then calculated during data analysis.

Current guidelines recommend maximization of NIV duration during the first 24 h, followed by tapering NIV according to gas exchange levels and tolerance in the next 2 to 3 days [11]. Thus, three temporal moments were defined: D1—the day of admission; D3—the 3rd day of NIV; and DoD—the day of discharge. For each of these days, we measured the average ΔPes.

We also obtained the number of hours of NIV and the mean IPAP during the first 24, 48, and 72 h after admission. Other measured variables were anthropometric data (age, BMI), ventilatory function (FEV1, FEV1/FVC), length of hospital stay (LOS), and the number of days of NIV.

The main objective of our study was to evaluate the safety and accuracy of the proposed method for Pes measurement in patients with ARF due to severe exacerbation of COPD requiring NIV in an RICU. Secondary objectives were to assess the impact of body position, body weight, and NIV on the variation of ΔPes.

### 2.4. Data Analysis

After recording the data in raw form, Pes and Paw curves were digitally processed for artefact removal. To this end, we performed primary data analysis using the GNU Octave software, an open-source version of MATLAB software (MathWorks, Natick, MA, USA).

We developed an original digital filter based on a direct analysis of the Pes recordings over time. Determining the minimum and maximum pressure points on the signal curve allows for an immediate evaluation of the parameters of a respiratory half-cycle—Δp (the amplitude of a half-cycle) and Δt (the duration of a half-cycle). We applied a Savitzky–Golay-type digital filter to achieve this while using a second-grade derivation and five or seven samples. First, we established the position at a time of the minimum and maximum points. Then, we determined the value of the pressure by choosing the closest value to the extremes of the curve. This procedure ensured the correct preservation of the amplitude of the respiratory cycles.

Once all the respiratory cycles were listed, we selected a series of criteria for recognizing and eliminating the artefacts (deviations from the mean amplitude of the Δp cycles). First, we calculated the Z score for the entire half-cycle. Then, high-amplitude artefacts were defined as those with a Z score for Δp greater than 0.95, while low-amplitude artefacts were those with a Z score for Δp lower than 0.5. However, in some cases, it was necessary to adjust the parameters manually based on a visual assessment of the eliminated cycles.

Finally, we calculated ΔPes and ΔPaw variation for each of the four recording stages and each time frame.

Statistical analysis was performed using SPSS version 20 (SPSS Inc., Chicago, IL, USA). We used the independent, paired *t*-test and ANOVA to calculate the differences between the recorded stages and time frames. The results were presented as mean difference (mean diff, standard deviation (Std. Dev.)), and 95% confidence interval (95% CI). The differences were statistically significant at *p* < 0.05. Given the sample of 15 patients, we considered a trend towards statistical significance at *p* < 0.1.

## 3. Results

Fifteen patients (8 men, mean age 65.1 years) were included in the study. We present their characteristics in Table 1.

During the entire hospital stay, no patients reported laryngeal or swallowing discomfort. We did not record any episodes of vomiting or nausea. All 15 patients were successfully ventilated non-invasively and discharged at home. Five of them (33%) further required home NIV. One patient refused to keep the esophageal catheter fitted after the first 48 h but was held in the study. In two patients, given their clinical severity, we could not record all the proposed Pes measurements.

### 3.1. Pes Measurement: Raw Results and Signal Analysis

**Figure 1:**
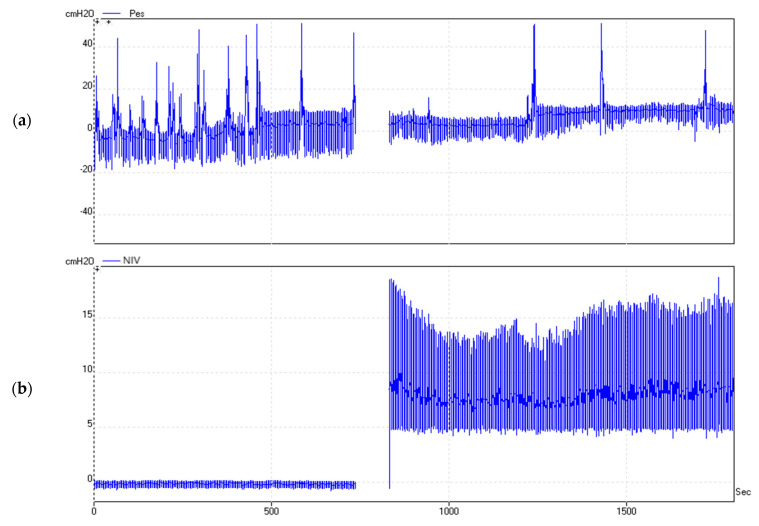
Real-time measurement of Pes (**a**) and Paw (**b**) for 30 min. Significant variation of Pes can be observed depending on the patient’s position and the presence of NIV. Paw, airway pressure measured at the mask level during noninvasive ventilation.

The duration of each recording was 30 min. An example is illustrated in Figure 1.

**Figure 2:**
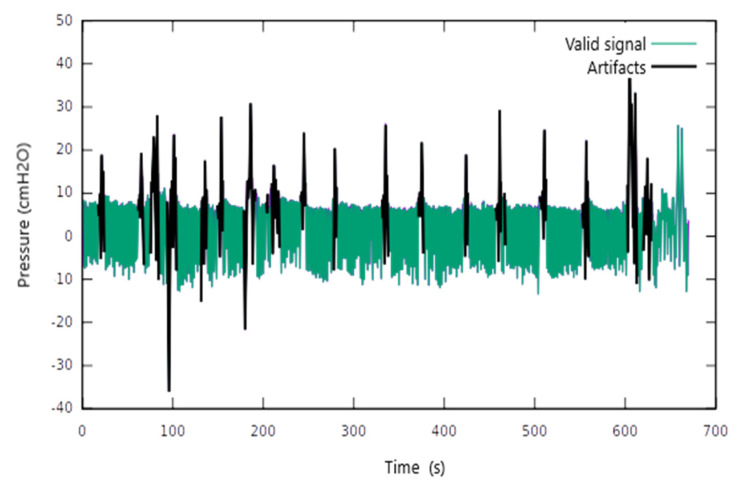
The application of the digital filter to remove the signal artefacts.

We identified signal areas with multiple artefacts. Disproportional oscillations of the Pes values were recorded in amplitude and duration (Figure 2). These artefacts were encountered regardless of the patient’s position or the presence of ventilator support. Most artefacts could be related to specific events such as swallowing, coughing, or the movement of the trunk. Consequently, we conducted artefact removal by applying the described digital filter. Figure 2 shows the remaining Pes signal after artefact removal.

### 3.2. Measurement Results for ∆Pes

Table 2 shows the values for ∆Pes (mean and interval) obtained after applying the digital filter.

### 3.3. Influence of Body Position on ∆Pes in the Absence of NIV

In the absence of NIV, ∆Pes values were higher in the supine position at D1, D3, and DoD. The maximum values for ∆Pes were recorded at D1 (mean ∆Pes 23.2 cm H_2_O) (Table 2). 

At D1, ∆Pes increased with a mean of 6.30 cm H_2_O (*p* < 0.01) when switching from the upright to the supine position. This variation of ∆Pes was correlated with the BMI (*p* = 0.07).

There was no significant change in ∆Pes between D3 and DoD regardless of the position

∆Pes increased when switching from the upright to the supine position with 3.71 cm H_2_O (*p* < 0.01) at D3 and with 3.44 cm H_2_O (*p* = 0.01) at DoD (Table 3).

In the upright position, we found a statistically significant decrease in mean ∆Pes between D1 and D3 (4.38, *p* < 0.07).

∆Pes decreased between D1 and DoD in the upright position with a mean of 3.47 (95% CI −0.47 −7.38, *p* = 0.02) cm H_2_O. The change was correlated with BMI (*p* < 0.01).

### 3.4. Influence of Ventilatory Support on ∆Pes

The addition of NIV led to a significant reduction of ∆Pes both at D1 and D3 (*p* < 0.01).

The addition of NIV at D1 reduced ∆Pes with an average of 5.18 cm H_2_O (*p* < 0.01) in the upright position and with 8.84 cm H_2_O (*p* < 0.01) in the supine position. Under ventilatory support, ∆Pes values remained higher in the supine position at D1 (mean ∆Pes 13.8 cm H_2_O) and D3 (mean ∆Pes 9.5 cm H_2_O).

The mean increase of ∆Pes between upright and supine positions during NIV was 0.94 cm H_2_O at D1 and 0.92 cm H_2_O at D3 (*p* < 0.01).

Table 3a,b show how ∆Pes reacts to the change in the patient’s position and the addition of NIV during the same day and how it varies between different days.

**Figure 3:**
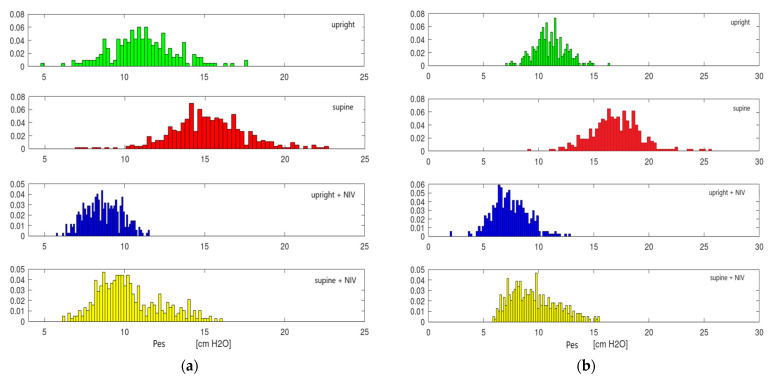
Variation of ∆Pes at D1 (**a**) and D3 (**b**) depends on the position and presence of NIV. Normal data distribution can be observed.

We illustrate an example of ∆Pes variation depending on the position and the presence of NIV in Figure 3.

## 4. Discussion

In 15 patients with AECOPD with AHRF that required NIV, we successfully measured Pes daily from admission until discharge. To our knowledge, this is the first study conducted in a respiratory intermediate care unit in patients with AECOPD.

Patients presented with a mean baseline value for ∆Pes of 23 cm H_2_O. However, this value was significantly reduced 24 h later.

In earlier studies, the measurement of Pes was mainly used for physiological observations on the effect of MV on respiratory mechanics. Even though the possibility of using Pes to guide the management of MV in intubated patients has been thoroughly studied, there are few data regarding its utility in patients with ARF during NIV [1]. Goldberg et al. used Pes measurement to evaluate the effect of CPAP in severe AECOPD with ARF in a small study that included ten patients. The application of CPAP was shown to reduce Pes swings and improve dyspnea [4]. The effect of different modes of NIV in patients with AECOPD with AHRF was further studied by Girault et al. and Wysocki et al. using ∆Pes, PTPes, and WOB measurements [5,6]. Chadda et al. evaluated the effect of CPAP vs. BPAP on WOB in patients with cardiogenic pulmonary oedema measuring both ∆Pes and PTPes [13]. Pes measurement was also used to assess the efficacy of NIV in the presence of large mask air leaks in patients with acute lung injury and AIDS [14]. All these studies are more than 20 years old and were conducted at a time when the application of NIV in ARF was an emerging technique.

More recent studies that included pediatric patients with ARF were conducted to evaluate the physiologic effect of different NIV modes on respiratory mechanics [15,16]. Pes measurement was also used to compare the impact of varying NIV interfaces on the respiratory pattern in healthy adults [17]. Grieco et al. also used Pes measurement for a physiological comparison between high-flow oxygen therapy and NIV in adult patients with acute hypoxemia [7].

Finally, the application of Pes measurement as a tool for guiding NIV management in patients with ARF has only recently been proposed. In a pilot study of 30 patients, Tonelli et al. found that a reduction in ∆Pes of 10 cm H_2_O or more after 2 h of NIV is a strong indicator of NIV success in patients with hypoxic de novo respiratory failure [8]. Mortamet et al. used Pes and PTPes as an indicator for initiating NIV but also guided NIV settings and duration in a study group of seven infants with AHRF [9].

Our study is part of a larger project aiming to use Pes measurement to guide NIV management in an acute setting. The first stage comprised developing and testing a protocol for Pes measurement in patients with ARF. This included the development of a digital analysis procedure that could allow for accurate Pes estimation after removing signal artefacts. To this goal, we conducted a study in an RICU environment with Pes measurements recorded throughout the hospital stay.

The Pes catheter was kept in place throughout the hospital stay, allowing for repeated daily measurements, including at discharge.

The variation of ∆Pes was correlated with the anthropometric parameters and body position (upright and supine position) even at admission during the acute phase of AECOPD.

The insertion of the Pes catheter did not pose significant difficulties even though it was highly dependent on patient cooperation. All patients presented with a high respiratory rate and increased respiratory effort at admission. Despite this, the insertion of the esophageal catheter did not pose any technical difficulties, was well tolerated, and was accepted by the patients, and in most cases, did not require more than 10 min of NIV break. No side effects (i.e., nausea, vomiting, dysphagia, etc.) were reported after the insertion of the catheter or at any time during the hospital stay. The presence of the catheter during NIV sessions did not lead to significantly increased air leaks. Air leaks increased between 15 and 20%, which did not exceed the NIV normal working parameters. Furthermore, no Pes signal artefacts could be attributed to the presence of NIV. Thus, we conclude that any NIV full face mask can be used during Pes measurement, and no special NIV equipment is needed during this procedure.

Measurements done in both positions have been well-tolerated regardless of the presence or absence of NIV. During visual inspection of the initially recorded signal, some observations were made regarding artefacts and Pes variation.

Isolated signal artefacts were reported in all patients, with some having a clear cause such as swallowing, coughing, or body movements. For example, swallowing led to a transitory increase in the absolute values of Pes of up to 20 cm H_2_O. Coughing led to a much more significant rise in Pes of up to 30 cm H_2_O measured from the mean ∆Pes. Some patients presented with additional artefacts during spontaneous breathing, for which we could not identify the clinical trigger. Interestingly, most of these unexplained artefacts disappeared during NIV. This could be related to either the variable elastance of the esophagus wall and possibly inadequate filling volume of the balloon or the esophageal contractions [18,19].

Baseline artefacts can increase the absolute baseline value of Pes above the pleural value [20]. Consequently, artefact removal is necessary to estimate transpulmonary lung pressures that can be used to personalize mechanical ventilation settings according to the patient’s respiratory mechanics [3]. The application of our developed digital filter managed to eliminate up to 95% of the recorded artefacts successfully.

In the absence of NIV, we observed increased Pes swings in all patients in supine positions. Again, these visual differences were more significant in obese patients.

Moreover, during the visual inspection, the difference in Pes swings between the two body positions was much lower while applying NIV.

We recorded the highest ∆Pes values during the first 1–2 h of admission, with a significant decrease after 24 h, which was correlated with clinical and ABG improvement. It is well-established that NIV failure usually occurs within the first 24 to 48 h after admission. Thus, early detection of NIV failure is crucial in improving the outcome of these patients [21], and Pes measurement could be an accurate indicator of early NIV failure [8].

∆Pes values in supine vs. upright positions were higher in the absence of NIV. This was previously described in healthy subjects [22] and is justified by the fact that in the supine position, respiratory muscle strain is increased through reduction of functional residual capacity, increase in elastic recoil of the thoracic cage, increase in abdominal pressure, and reduction of lung compliance [23,24].

During NIV, the difference in ∆Pes between positions was reduced, and this effect was seen regardless of the recording time. Lower negative Pes swings correspond to lower WOB, which was expected under NIV [25]. Furthermore, we may assume that given that an adaptive pressure mode was used (VAPS mode), this may have contributed to maintaining relative constant ∆Pes despite changing body position without modifying NIV parameters.

A significant reduction in ∆Pes was observed within the first 24 h of admission regardless of the patient’s position. After 24 h, no further statistically significant decrease was seen. Furthermore, the mean duration of NIV was 4.5 days. This could suggest that in severe AECOPD patients, after one day of NIV, lung mechanics do not further improve but are more likely maintained at a constant level under NIV until further improvement in the dynamic lung hyperinflation is achieved. The existing published data do not offer a clear recommendation regarding the optimal duration of NIV in these patients with severe AECOPD. Thus, the Pes measurement, in association with other indicators, could provide further insight regarding the optimal time of NIV withdrawal [26].

Regarding the relationship between BMI and ∆Pes variation, it has been shown that obesity reduces lung volumes, increases resistance, and reduces respiratory system compliance [27]. However, we found only one study that investigated the relationship between NIV, body position, and BMI and the variation of ∆Pes. Porta et al. measured ∆Pes and PTPes in a group of 12 patients with stable COPD (at least four weeks after an exacerbation). Similar to our study, measurements were performed with the patients in different positions with or without NIV support. Their results showed that adding NIV reduced ∆Pes from 14 ± 4.8 to 6.2 ± 3.5 cm H_2_O, with this reduction being BMI-dependent. However, only one measurement was performed on each patient [28]. We detected a similar, BMI-dependent fall in ∆Pes on day 1 (i.e., 8 ± 2.72 cm H_2_O) while the patient was sitting.

Moreover, mean values of ∆Pes under NIV on day 1 were higher than those recorded by Porta et al. (12 and 14 cm H_2_O vs. 8.8 and 9.4 cm H_2_O, respectively). This could be related to our recordings being performed in patients with severe AECOPD. In our study, ∆Pes was only partially dependent on BMI. This could be explained by the fact that the mechanism of ARF in AECOPD is mainly related to dynamic hyperinflation, ventilation/perfusion mismatching, and an increase in the physiological dead space [29]. Owens et al. found similar results when measuring absolute values of Pes in lean and obese subjects with no respiratory disease. They noticed greater Pes values in overweight and obese subjects, and changes in position (seated vs. supine) were similar between the two groups [30]. 

Our study has limitations. Unlike other studies, the absence of a pneumatograph and the use of an esophageal catheter instead of a gastro-esophageal catheter did not allow us to measure respiratory flow, PTPes, and transdiaphragmatic pressure. In addition, the number of patients included in the study was small, and the fact that we could not measure Pes more than once per day did not allow us to have a detailed view of Pes evolution, especially within the first 24 h. Furthermore, signal analysis was conducted during a later stage. In the future, we plan to develop a neural network that can automatically predict (in real time) acceptable values using our developed signal analysis algorithm.

Regardless of these limitations, our study shows that the proposed protocol for measuring and analyzing Pes is accurate and well-tolerated by patients with AECOPD requiring NIV.

## 5. Conclusions

The measurement of ∆Pes allows for easy and safe quantification of the respiratory effort and its response to the application of NIV during AHRF in AECOPD. However, signal artefact removal is necessary for an accurate estimation of ∆Pes.

In patients with AECOPD requiring NIV, ∆Pes reduction is most significant within the first 24 h of admission. These patients have increased ∆Pes in the supine position, especially if they are obese.

The measurement of ∆Pes could prove a valuable tool in the management of NIV in patients with ARF.

## Figures and Tables

**Table 1:** Patient characteristics.

Variable	Mean	Interval (Min–Max)
Age	65.1	52–79
BMI	29.2	18–38
mIPAP	18.2	11–27
FEV1 % pred	28.2	19–51
dVNI-72 h (h)	24.2	8–41
Days of NIV	4.6	2–9
LOS	8	4–12

Legend: BMI, body mass index; mIPAP, mean inspiratory pressure in the first 72 h; FEV1, forced expiratory volume in the 1 s; dVNI-72 h, hours of NIV in the first 72 h of hospitalization; LOS, length of hospital stay.

**Table 2:** Recorded mean ∆Pes values.

Time	Position ± NIV	∆Pes	
		Mean	Interval
D1	Upright	17.7	9.1–28.3
	Supine	23.2	15.2–50.8
	Upright + NIV	12.0	5.3–27.2
	Supine + NIV	13.8	6.0–40
D3	Upright	11.7	4.0–17.4
	Supine	15.2	5.3–24.1
	Upright + NIV	8.5	3.4–15.8
	Supine + NIV	9.5	4.5–22
DoD	Upright	13.0	6.0–19.5
	Supine	17.3	8.1–29.4

Legend: D1, 1 day of admission; D3, 3rd day of admission; DoD, day of discharge.

**Table 3:** (**a**) The intraday variation of mean ∆Pes (i.e., the mean difference of ∆Pes) relative to the position and the presence of NIV support and its relationship with BMI. (**b**) The interday variation of mean ∆Pes (i.e., the mean difference of ∆Pes) relative to the position and its relationship with BMI in the absence of NIV.

(**a**)								
	**Patient’s Position and NIV Support**		**Mean Difference**	**Std. Dev.**	**CI 95%**		*p* *	BMI ** (*p*)
					**Min**	**Max**		
D1	Upright	Supine	−6.30	6.11	−10.41	−2.20	<0.01	0.07
	Upright	Upright NIV	5.18	4.12	2.69	7.67	<0.01	
	Supine	Supine NIV	8.84	3.61	6.55	11.14	<0.01	
	Upright NIV	Supine NIV	−0.94	2.39	−2.47	0.57	ns	
D3	Upright	Supine	−3.71	3.44	−6.02	−1.39	<0.01	
	Upright	Upright NIV	3.82	5.09	0.18	7.46	0.04	
	Supine	Supine NIV	6.75	7.22	2.16	11.33	0.01	
	Upright NIV	Supine NIV	−0.92	2.97	−2.81	0.96	ns	
DoD	Upright	Supine	−3.44	3.07	−6.29	−0.60	0.02	
(**b**)								
	**Patient’s Position**		**Mean Difference**	**Std. Dev.**	**CI 95%**		*p* *	BMI ** **(*p*)**
					**Min**	**Max**		
D1–D3	D1 upright	D3 upright	4.38	7.19	−0.45	9.21	*0.07*	
	D1 supine	D3 supine	7.13	12.30	−2.33	16.58	ns	
D1–DoD	D1 upright	DoD upright	3.47	5.09	−0.45	7.38	0.02	<0.01
	D1 supine	DoD supine	2.51	5.31	−2.40	7.42	ns	
D3–DoD	D3 upright	DoD upright	0.11	0.91	−0.73	0.95	ns	
	D3 supine	DoD supine	−1.31	4.93	−6.49	3.86	ns	

Legend: D1, 1 day of admission; D3, 3rd day of admission; Dx, last day of NIV; DoD, the day of discharge; * *t*-test; ** ANOVA. Data are expressed as mean difference (mean diff, standard deviation (Dev. Std), 95% confi-dence interval (95% CI), *p* for *t*-test and p for Anova test (BMI (*p*)).

## Data Availability

The data presented in this study are available on request from the corresponding author. The data are not publicly available due to privacy and ethical concerns.
